# Canocapavir Is a Novel Capsid Assembly Modulator Inducing a Conformational Change of the Linker Region of HBV Core Protein

**DOI:** 10.3390/v15051195

**Published:** 2023-05-18

**Authors:** Yuan Zheng, Le Yang, Lin Yu, Yuanfei Zhu, Yang Wu, Zhijun Zhang, Tian Xia, Qiang Deng

**Affiliations:** 1 Key Laboratory of Medical Molecular Virology (MOE/NHC/CAMS), Shanghai Frontiers Science Center of Pathogenic Microorganisms and Infection, School of Basic Medical Sciences, Fudan University, Shanghai 200032, China; 19211010056@fudan.edu.cn (Y.Z.); 21211010060@m.fudan.edu.cn (L.Y.); 20111010069@fudan.edu.cn (L.Y.); zhuyuanfei@fudan.edu.cn (Y.Z.); yangwu@fudan.edu.cn (Y.W.); 2 Shanghai Institute of Infectious Disease and Biosecurity, Shanghai 200032, China; 3 Shanghai Zhimeng Biopharma, Inc., 1976 Gaoke Middle Road, Suite A-302, Pudong District, Shanghai 201210, China; zzhang@corebiopharma.com

**Keywords:** hepatitis B virus, hepatitis B virus core protein, core protein allosteric modulator, capsid assembly, viral particle egress, allosteric effect

## Abstract

Canocapavir is a novel antiviral agent with characteristics of core protein allosteric modulators (CpAMs) that is currently in a phase II clinical trial for treatment of hepatitis B virus (HBV) infection. Herein, we show that Canocapavir prevented the encapsidation of HBV pregenomic RNA and increased the accumulation of cytoplasmic empty capsids, presumably by targeting the hydrophobic pocket at the dimer-dimer interface of HBV core protein (HBc). Canocapavir treatment markedly reduced the egress of naked capsids, which could be reversed by Alix overexpression through a mechanism other than direct association of Alix with HBc. Moreover, Canocapavir interfered with the interaction between HBc and HBV large surface protein, resulting in diminished production of empty virions. Of particular note, Canocapavir induced a conformational change of capsids, with the C-terminus of HBc linker region fully exposed on the exterior of capsids. We posit that the allosteric effect may have great importance in the anti-HBV activity of Canocapavir, given the emerging virological significance of HBc linker region. In support of this notion, the mutation at HBc V124W typically recapitulated the conformational change of the empty capsid with aberrant cytoplasmic accumulation. Collectively, our results indicate Canocapavir as a mechanistically distinct type of CpAMs against HBV infection.

## 1. Introduction

Hepatitis B virus (HBV) is a small, enveloped DNA virus that attacks the liver and can cause both acute and chronic hepatitis [1]. Despite being a DNA virus, HBV replicates its genome through the protein-primed reverse transcription of pregenomic RNA (pgRNA) in the nucleocapsids, where pgRNA is converted to rcDNA catalyzed by the reverse transcriptase (RT) protein. rcDNA-containing nucleocapsids can be enveloped by three viral surface proteins, large, middle, and small hepatitis B surface proteins (LHBs, MHBs, and SHBs), which are all encoded by the envelope gene and secreted as complete virions. Additionally, mature nucleocapsids can be imported into the nucleus via the so-called intracellular amplification pathway to replenish the intracellular cccDNA pool [2,3]. The production of virions is always accompanied by the formation of subviral particles [4]. The classical Australian antigen is a spherical or tubular particle with a diameter of 22 nm, containing only viral envelope proteins and found in the blood of infected individuals at up to 100,000-fold in excess over the complete virions. Nonenveloped capsids (i.e., naked capsids) have been found in the cell culture of HBV-replicating cells in larger amounts than complete virions and also in the blood of infected patients in the form of capsid-antibody complexes [5]. Additionally, HBV secretes empty or genome-free virions that only contain the envelope and capsid, with levels 100-fold higher than those of complete virions both in vitro and in vivo [6].

HBV core protein (HBc), a small polypeptide of 183 or 185 amino acid residues depending on different HBV genotypes, consists of an N-terminal domain (NTD) and a C-terminal domain (CTD) connected by a short linker peptide with conserved sequence [7]. The 140 residues of NTD form the α helix-rich assembly domain, capable of self-assembling into icosahedral capsids at sufficiently high concentrations [8,9]. The arginine rich CTD contains several phosphorylation sites. HBc CTD is hyperphosphorylated in HBc dimers, and its dephosphorylation promotes pgRNA packaging [10,11,12]. It is reported that host protein phosphatase 1 (PP1) can be detected in mature HBV virions and is presumably involved in the regulation of HBc dephosphorylation and pgRNA packaging [13]. Recent studies also demonstrate that HBc linker has a significant impact on the pgRNA encapsidation, DNA synthesis, and regulates the secretion of viral particles [14,15,16,17]. Specific mutations of the residues in the HBc linker region inhibit the secretion of empty virions without affecting the production of rcDNA-containing virions. Additionally, the sequence of the HBc linker is highly matched with a consensus binding motif for the protein phosphatase 2A (PP2A) regulatory subunit, indicating that the HBc linker can regulate the phosphorylation state of HBc by recruiting PP2A [15].

Core protein allosteric modulators (CpAMs) interfere with the process of HBV nucleocapsid assembly by binding at the hydrophobic pocket between the dimer–dimer interface of HBc subunits and are a promising antiviral drug candidates developed in recent years. Current CpAMs fall into two types; While type I CpAMs (also known as CAM-A), such as Bay 41-4109 and GLS-4, misdirect core protein assembly to form aberrant non-capsid polymers, type II CpAMs (also called CAM-E), such as NVR3-778 and JNJ-6379, induce the formation of morphologically normal capsids devoid of viral pgRNA and polymerase [18]. Herein, we report that Canocapavir, a new type of pyrazole compound [19], inhibits HBV replication and promotes the formation of intracellular empty capsids by targeting the hydrophobic pocket at the dimer-dimer interface of HBc. It is particularly interesting that Canocapavir inhibits the secretion and envelopment of viral particles, presumably by exerting an allosteric effect on the HBc linker region. Our study thus identifies a previously unreported mechanism for the anti-HBV activity of CpAMs and indicates Canocapavir as a distinct type of CpAMs against HBV infection. 

## 2. Materials and Methods

### 2.1. Cell Culture and Antiviral Compounds

HepG2, Huh7, HepG2.2.15, and HEK 293T cells were maintained in Dulbecco’s modified Eagle medium (DMEM) supplemented with 10% fetal bovine serum, 100 U/mL penicillin, and 100 µg/mL streptomycin. HepAD38^Tet-off^ cell line was maintained in DMEM/F12 medium supplemented with 10% fetal bovine serum, 100 U/mL penicillin, 100 µg/mL streptomycin, 1 µg/mL tetracycline, and 400 µg/mL G418. Removal of tetracycline from the culture medium will initiate HBV pgRNA transcription and DNA replication. HepG2-HBc^Tet-on^ cells were derived from HepG2 cells and expressed HBc under the Tet-on promoter. In this study, Canocapavir was provided by Shanghai Zhimeng Biopharma Inc.; ETV, Bay 41-4109, AB-423, JNJ-632, and JNJ-6379 were purchased from MCE.

### 2.2. DNA Constructs

pcDNA3.1-HBc, -HBc aa1–140, -HBc aa1–144, -HBc aa1–145, -HBc aa1–147, and -HBc aa1–155 were constructed by inserting into the pcDNA3.1 vector between the *Hind*III and *EcoR*I site with the full-length or truncated HBc. pcDNA3.1-HBc derived plasmids expressing HBc mutants were generated via PCR mutagenesis. pHBV1.1 encodes a 1.1-overlength copy of the HBV genome (serotype: ayw), with pgRNA driven by the cytomegalovirus (CMV) immediate-early promotor. pHBV1.1-Δpol was derived from pHBV1.1 and defective in HBV polymerase expression and generated by displacing the starting codon of the polymerase reading frame with ACG without altering the amino acids of HBc. To generate an Alix expression construct with a C-terminal HA tag, the DNA fragment was amplified by PCR using pWPI-Alix-mcherry as a template, which was kindly provided by Prof. Gang Long (Fudan University, China) and inserted into the pCDH vector between the *EcoR*I and *BamH*I site. pCMV-Flag-LHBs expressed the full length of HBV large surface protein with an N-terminal Flag tag and was kindly provided by Prof. Youhua Xie (Fudan University, China).

### 2.3. Antibodies 

Rabbit polyclonal anti-HBc antibody is from DAKO (B0586). For the detection of capsids, mouse monoclonal anti-HBc C1 (ab8637) was purchased from Abcam, and mouse monoclonal anti-HBc 2A7 was kindly provided by Prof. Quan Yuan (Xiamen University, China). For the immunoprecipitation of HBV virions, rabbit anti-HBs (NB100-62652, Novus, Centennial, CO, USA) and rabbit anti-preS1 antibodies (kindly provided by Prof. Shuping Tong, Brown University, USA) were used. For immunofluorescence staining, commercially available rabbit polyclonal anti-Alix (12422-1-AP, Proteintech, Chicago, IL, USA), and rabbit polyclonal anti-HBc (0282, Long Island Antibody, Shanghai, China) were used. Other commercially available antibodies are as follows: mouse anti-β-tubulin (M20005, Abmart, Shanghai, China), mouse anti-β-actin (T40104, Abmart, Shanghai, China), and rabbit anti-Flag (14793S, Cell Signaling Technology, Boston, MA, USA).

### 2.4. siRNA and Transfection

Transient transfection of cells was performed by mixing the plasmids and Neofect™ DNA transfection reagent according to the manufacturer’s instructions. To inhibit the expression of Alix, siRNA duplexes targeting the sequence (GGCACAGGCTCAAGAAGTA) of Alix were used (RiboBIO, Guangzhou, China). Briefly, 3 × 10^5^ cells per well of a 12-well plate were transfected with 15 pmol siRNA and 4.5 μl Lipofectamine RNAiMAX transfection reagent (13778, Thermo Fisher Scientific, Waltham, MA, USA) according to the protocol of the supplier. As a control, a nonsense siRNA with no known homology to the mammalian gene was used (RiboBIO, Guangzhou, China). After 36 h, cells were retransfected with plasmid DNA with Neofect™ DNA transfection reagent and harvested after an additional 48 h.

### 2.5. Southern Blot Analysis of HBV Replicative Mediates

HBV core DNA was extracted as previously described, with minor modifications [20]. Briefly, cells were lysed in 200 µL lysis buffer containing 0.5% NP-40, then incubated with Mung Bean Nuclease (M0250S, New England Biolabs, Ipswich, MA, USA) and DNase I (EN0521, Sangon Biotech, Shanghai, China) to remove the input plasmid DNA. Viral DNA was ethanol precipitated after protease K (Calbiochem, San Diego, CA, USA) digestion in the presence of 0.5% SDS at 55 °C overnight. Purified DNA was dissolved in 16 µL double-distilled water and separated by 1% agarose gel. Following overnight transfer to nylon membrane, the blot was hybridized with a Digoxigenin-labeled HBV-specific probe.

### 2.6. Analysis of Viral Particles

Intracellular HBV capsids and associated viral DNA were analyzed by a native agarose gel electrophoresis-based assay [21]. Culture supernatant containing virions and naked capsids was concentrated with polyethylene glycol precipitation and resolved in 25 µL TNE buffer containing 0.05% β-ME. To detect HBV surface and core antigens, the membrane was soaked in phosphate-buffered saline containing 2.5% formaldehyde at room temperature for 10 min. After being briefly rinsed with water, the membrane was then soaked in 50% methanol at room temperature for 30 min and washed three times with double-distilled water. The next steps were paralleled with the particle gel analysis.

### 2.7. Western Blot Assay

Cells in the wells of the 12-well plate were washed with phosphate-buffered saline twice, then lysed in 100 µL 1 × SDS loading buffer. A total of 10 µL of the cell lysate was resolved by SDS-PAGE and transferred onto a polyvinylidene difluoride (PVDF) membrane (Merke Millipore, Germany; 0.2 μm). The membrane was blocked with tris-buffered saline containing 0.1% Tween and 5% nonfat dry milk and probed with the appropriate primary antibody.

### 2.8. Ultracentrifugation of HBV Capsids

Cells in a 10 cm plate were lysed in 2 mL of cell lysis buffer (10 mM Tris-HCl, pH7.5, 1 mM EDTA, 0.5% NP40) at room temperature for 10 min. The cell debris was cleared by centrifugation at 12,000× *g* for 10 min at 4 °C. The supernatant was loaded onto a 30% sucrose cushion and centrifuged at 46, 000 rpm for 3.5 h (Beckman, Indianapolis, IN, USA; SW55 rotor). The pellet was dissolved in 200 µL TNE buffer containing protease inhibitor (5892953001, Roche, Basel, Switzerland) overnight, then loaded onto a 15–50% linear sucrose gradient in TNE buffer and centrifuged at 27,000 rpm for 8 h (Beckman, Indianapolis, IN, USA; SW55 rotor). Fractions (250 µL/each) were collected from the top of the centrifugation tube. A total of 25 µL of each fraction was applied to agarose gel electrophoresis or SDS-PAGE to detect viral capsids or HBc.

### 2.9. Immunofluorescence Staining

Huh7 cells were washed and fixed in 4% paraformaldehyde for 10 min at room temperature. Fixed cells were then permeabilized with 0.1% Triton X-100 in PBS for 30 min at room temperature. After being blocked in PBS containing 20% goat serum, cells were incubated with the indicated primary antibodies for 1 h, rinsed with PBS, then incubated with AlexaFluor-tagged secondary antibodies (Thermo Fisher Scientific, Waltham, MA, USA) for 1 h. Cells were then stained with DAPI (C1002, Beyotime, Shanghai, China) to show the nuclei.

### 2.10. Immunoprecipitation of Virions in Supernatant

Empty virions were immunoprecipitated from 1.5 mL precleared culture supernatant on day 4 post-transfection by adding 4 µL rabbit polyclonal anti-preS1 or a combination of 4 µL rabbit polyclonal anti-preS1 and 2 µL rabbit anti-S pre-conjugated to 25 µL protein A/G plus agarose (sc-2003, Santa Cruz, Dallas, TX, USA), respectively. The immune complexes were washed five times with tris-buffered saline containing 0.5% Tween-20 prior to SDS–PAGE and immunoblotting.

### 2.11. Co-Immunoprecipitation Assay

HEK 293T cells were lysed at 4 °C for 30 min in lysis buffer (1 % Triton X-100, 50 mM Tris-HCl pH 7.4, 150 mM NaCl, and 1 mM EDTA) freshly supplemented with complete EDTA-free protease inhibitors (5892953001, Roche, Switzerland). Cell debris was removed by centrifugation at 16,000× *g* for 10 min. The clarified supernatants were then incubated with rabbit monoclonal anti-Flag pre-conjugated to 25 µL protein A/G plus agarose beads and rotated at 4 °C overnight. The immune complexes were washed five times prior to SDS–PAGE and immunoblotting.

### 2.12. Molecule Docking Analysis

The HBc sequence used in the simulation was downloaded from the Protein Data Bank, identification number 6J10. The structure of Canocapavir was prepared with ChemDraw and Chem3D software. The ligand and protein structure were subjected to energy minimization and then for docking analysis using Autodock Vina. 

### 2.13. ELISA

HBsAg and LHBsAg in cell culture supernatant were detected using commercial enzyme-linked immunosorbent assay kits (KHB, Shanghai, China) according to the manufacturer’s instructions.

### 2.14. Real-Time PCR HBV 

Core DNAs were obtained as described [20]. Absolute real-time PCR was quantified by PowerTrack^TM^ SYBR Green Master Mix (A46012, Thermo Fisher Scientific, Waltham, MA, USA). Total RNAs were extracted using TRIzol Reagent (Invitrogen, Carlsbad, CA, USA) according to the manufacturer’s instructions. First-strand cDNA was synthesized from 3 μg of RNA using the FastKing-RT SuperMix kit (KR118, TIANGEN, Beijing, China). Relative RNA expression levels were quantified by PowerTrack^TM^ SYBR Green Master Mix, with relative amounts calculated using the 2^−ΔΔCt^ method (normalized to GAPDH).

### 2.15. Statistical Analysis

Data were expressed as means ± standard errors of the mean. Unpaired Student *t* tests were performed with GraphPad Prism software.

## 3. Results

### 3.1. Characterization of the Antiviral Activity of Canocapavir

**Figure 1:**
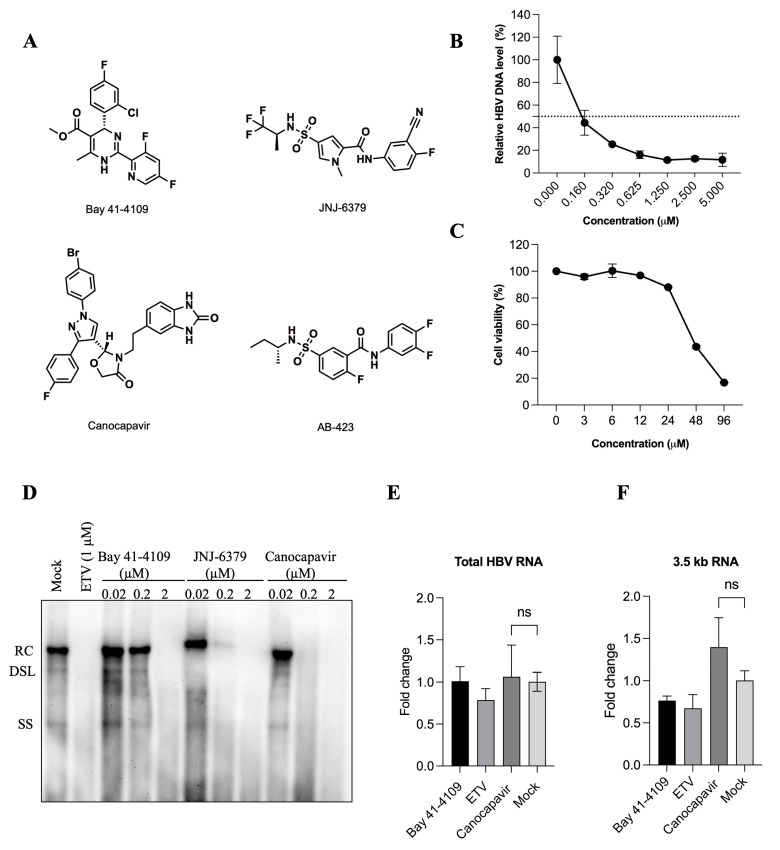
Canocapavir inhibits HBV replication effectively. (**A**) Chemical structures of Canocapavir and three reference CpAMs (Bay 41-4109, JNJ-6379, and AB-423) are presented. (**B**,**C**) Antiviral activity in HepAD38 cell line and cellular toxicity of Canocapavir. HBc and viral replication were induced in HepAD38 cells after the withdrawal of tetracycline from the culture medium. Cells were treated with Canocapavir at indicated concentrations for 2 days. Capsid-associated HBV DNA was quantified by real-time PCR. Data were expressed as the percentage of the mock-treated controls. The means and standard deviations (*n* = 3) were plotted. The cytotoxicity was determined by an MTT assay. (**D**) A total of 8 h after transfection with pHBV1.1, HepG2 cells were treated with ETV, Bay 41-4109, JNJ-6379, or Canocapavir for 4 days at indicated concentrations. HBV DNA replicative intermediates were determined by Southern blotting. RC, relaxed circular DNA; DSL, double-stranded linear DNA; SS, single-stranded linear DNA. (**E**,**F**) HepAD38 ^Tet-off^ cells were mock-treated or treated with the indicated concentrations of Bay 41-4109, ETV, or Canocapavir for 3 days. Total HBV RNA and the 3.5-kb RNA species were quantified by reverse transcription real-time PCR. ns, not significant.

Canocapavir is a new type of capsid assembly modulator. Distinct from the current type I and type II CpAMs, it has a pyrazole chemical structure (Figure 1A). In a HepAD38 cell line that replicates HBV in a Tet-off manner, Canocapavir inhibited HBV replication with a 50% effective concentration (EC50) of 0.1185 μM as determined by quantitative PCR without causing significant cytotoxicity up to 24 μM (Figure 1B,C). The antiviral potency of Canocapavir was further examined in HepG2 cells transiently transfected with a plasmid bearing 1.1-mer overlength HBV genome (i.e., pHBV1.1). Southern blotting indicated that Canocapavir inhibited HBV replication effectively in a dose-dependent manner, with similar results observed in cells treated with nucleoside analogue entecavir (ETV), type I CpAM Bay 41-4109, or type II CpAM JNJ-6379 (Figure 1D). Through analysis by quantitative reverse transcription real-time PCR assay, both total HBV RNAs and 3.5-kb RNA levels had no obvious alteration, suggesting that Canocapavir did not affect the viral transcription (Figure 1E,F).

### 3.2. Canocapavir Promotes Cytoplasmic Accumulation of Empty Nucleocapsids

**Figure 2:**
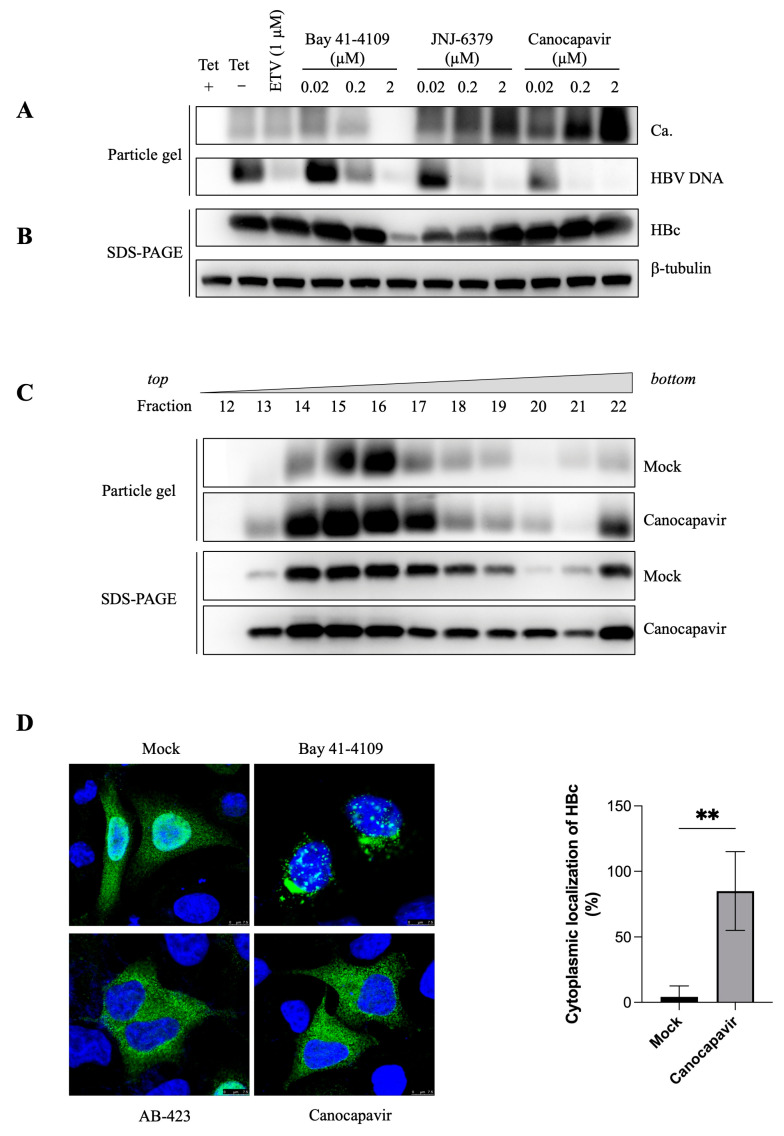
Canocapavir increases the accumulation of intracellular capsids devoid of the HBV genome. (**A**,**B**) HepAD38^Tet-off^ cells were mock-treated or treated with the indicated concentrations of ETV, Bay 41-4109, JNJ-6379, or Canocapavir for 6 days. Intracellular capsids were determined by immunoblotting with anti-HBc (C1) in a particle gel assay, with capsid-associated nucleic acids measured by hybridization with a DIG-labeled HBV-specific probe. The level of total HBc was determined by Western blotting with a mouse monoclonal antibody (2A7). Tubulin served as the loading control. (**C**) HepAD38^Tet-off^ cells were treated with 2 µM Canocapavir for 6 days. Intracellular capsids were sedimented on a sucrose gradient (15−50%) with a Beckman SW55 rotor. Twenty-two equal-volume fractions were collected from the top of the tube. Samples from each fraction were applied to Western blotting with anti-HBc (2A7), with capsids subjected to a particle gel assay and determined by anti-HBc (C1). (**D**) Canocapavir treatment abolished the nuclear distribution of HBc. Huh 7 cells with transient expression of HBc were left untreated or treated with 2 µM of Bay 41-4109, AB-423, or Canocapavir for 2 days. Cells were then stained with a rabbit polyclonal anti-HBc (Long Island) and a secondary Alexa Fluor 488-conjugated goat anti-rabbit antibody for confocal microscopy. Bar, 7.5 μm. Blue, DAPI; Green, HBc. Right, the percentage of cytoplasmic localization of HBc was calculated. ** (*p* < 0.05) Ca., Capsid.

To assess its effect on the capsid assembly, HepAD38^Tet-off^ cells were treated with Canocapavir, and ETV, Bay 41-4109, or JNJ-6379. As shown in Figure 2A,B, ETV had limited effect on the HBc expression and capsid assembly. In agreement with previous observations [22], Bay 41-4109 abolished capsid assembly and increased the propensity for the formation of non-capsid polymers dose-dependently. In contrast, Canocapavir substantially increased capsid formation without affecting intracellular HBc levels. DNA hybridization showed that all the CpAMs tested prevented the encapsidation of the HBV genome dose-dependently. ETV did not impair the encapsidation of pgRNA but inhibited the synthesis of HBV DNA, resulting in a weaker hybridization signal. Intriguingly, JNJ-6379 also increased the formation of the intracellular capsid, but this effect was less prominent as compared to the treatment with Canocapavir. Canocapavir treatment did not significantly alter the mobility of capsids in the native agarose gel electrophoresis. In addition, sucrose density gradient centrifugation showed that the capsids from Canocapavir-treated cells sedimented at a similar velocity compared with those from mock-treated cells and peaked at fractions 14, 15, and 16, respectively (Figure 2C). The capsid formed in the presence of Canocapavir was morphological normal, as evidenced by the images of transmission electron microscopy (EASL 2019, by Shanghai Zhimeng Biopharma, Inc.).

The arginine-rich CTD of HBc, of which the amino sequence resembles the structural features of known Serine and Arginine-rich (SR) proteins or nuclear localization signals (NLS), regulates the subcellular distribution of HBc [23]. Using immunofluorescence staining analysis, we explored the subcellular distribution of HBc in the cells treated with Bay 41-4109, sulfamoylbenzamide (SBA)-derivative AB-423, and Canocapavir, respectively (Figure 2D). As anticipated, Bay 41-4109 induced a remarkable perinuclear clustering of HBc-positive puncta, which was consistent with the formation of aberrant HBc polymers destinated for lysosomal traffic and degradation [24]. Different from the cell-wide dispersion of HBc in mock-treated cells, HBc was localized predominantly in the cytoplasm in both Canocapavir and AB-423 -treated cells. 

### 3.3. Overexpressing Alix Abolishes the Inhibitory Effect of Canocapavir on Naked Capsid Egress

**Figure 3:**
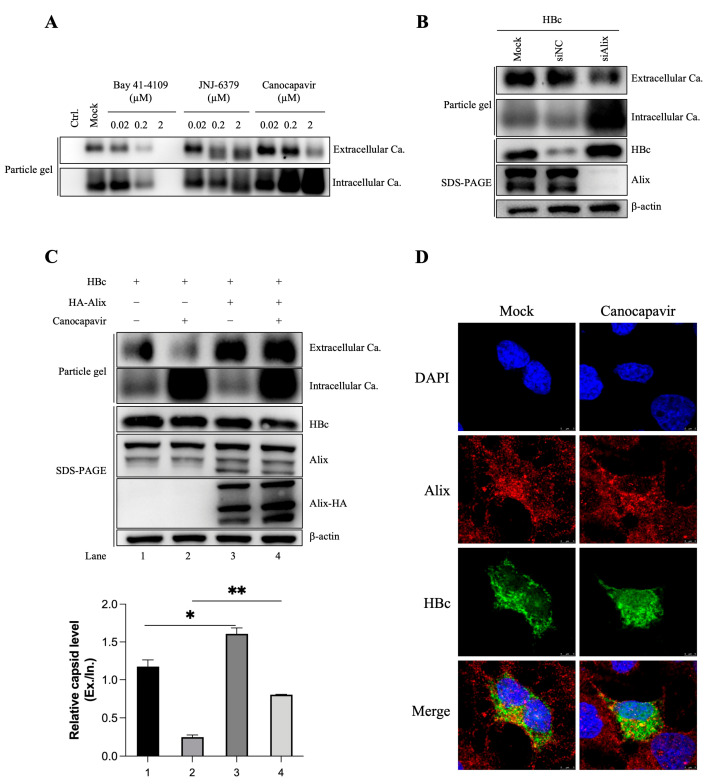
Canocapavir reduces the secretion of naked capsids, which can be reversed by Alix overexpression. (**A**) HepG2 cells with transient expression of HBc were left untreated or treated with the indicated concentrations of Bay 41-4109, JNJ-6379, or Canocapavir for 2 days. Naked capsids in cell lysates and concentrated cell culture media were resolved by native gel electrophoresis and subjected to immunoblotting with anti-HBc C1. (**B**) HepG2 cells were mock-transfected or transfected with Alix-specific siRNA for 36 h, followed by transfection with an HBc expression plasmid and cultured for another 48 h. Capsids in cell lysates and culture medium were assayed as depicted in (**A**). (**C**) HepG2 cells with exogenous co-expression of HBc and Alix were mock-treated or treated with 2 μM Canocapavir for 2 days, with capsids in cell lysates and culture media assayed by immunoblotting (upper panel). The relative density of extracellular capsids was calculated with data normalized to the level of intracellular capsids (bottom panel). * (*p* < 0.05); ** (*p* < 0.01). (**D**) Huh 7 cells transfected with pHBV-1.1 were left untreated or treated with 2 μM Canocapavir for 2 days and then stained with a mouse anti-HBc (2A7) [28] and a rabbit anti-Alix for immunofluorescence confocal microscopy. Alexa Fluor 488 conjugated goat anti-mouse antibody and Alexa Fluor 555 conjugated goat anti-rabbit antibody were secondary antibodies. Blue, DAPI; Red, Alix; Green, HBc. Bar, 5 μm.

HBV virions exploit the endosomal sorting complexes required for transport (ESCRT) pathway and multivesicular bodies (MVBs) for budding [25,26]. In addition, large amounts of naked capsids have been found to be noncytolytically released into the cell culture of HBV-replicating cells. In HepG2 cells transiently transfected with plasmids encoding HBc, Canocapavir treatment led to the remarkable accumulation of intracellular capsids, concomitant with diminished amounts of naked capsids in the supernatant (Figure 3A). A previous study identified Alix, a multifunctional protein with a key role in membrane biology, as a regulator of naked capsid budding [27]. Using small interfering RNAs (siRNAs)-mediated knockdown strategy, we observed considerably increased amounts of HBc and intracellular capsids upon the depletion of Alix, in accordance with the reduced level of the extracellular capsids, which was similar to that observed in Canocapavir-treated cells (Figure 3B). Intriguingly, overexpression of Alix resulted in slightly increased extracellular capsid levels in mock-treated cells and overwhelmingly reversed the inhibitory effect of Canocapavir on naked capsid secretion (Figure 3C).

Alix consists of an N-terminal boomerang-shaped Bro1 domain, a central V domain, and a C-terminal proline-rich region (PRR), with the Bro1 domain being reported to interact with HBc, driving the release of naked capsids [27]. To investigate whether Canocapavir interferes with the interaction between Alix and HBc, HBc and HA-tagged Alix, or the Bro1 domain, were co-expressed in Huh 7 cells. However, we did not observe appreciable interaction of HBc with Alix or its Bro1 domain in co-IP assays (Figure S1). In Huh 7 cells expressing HBc, the immunofluorescence staining did not show that Canocapavir treatment notably altered colocalization of HBc and endogenous Alix (Figure 3D). Collectively, our data show that Alix can reverse the inhibitory effect of Canocapavir on the budding of naked capsids through a mechanism other than direct association of Alix with HBc.

### 3.4. Canocapavir Inhibits HBV Envelopment by Interrupting the Interaction between HBc and LHBs

**Figure 4:**
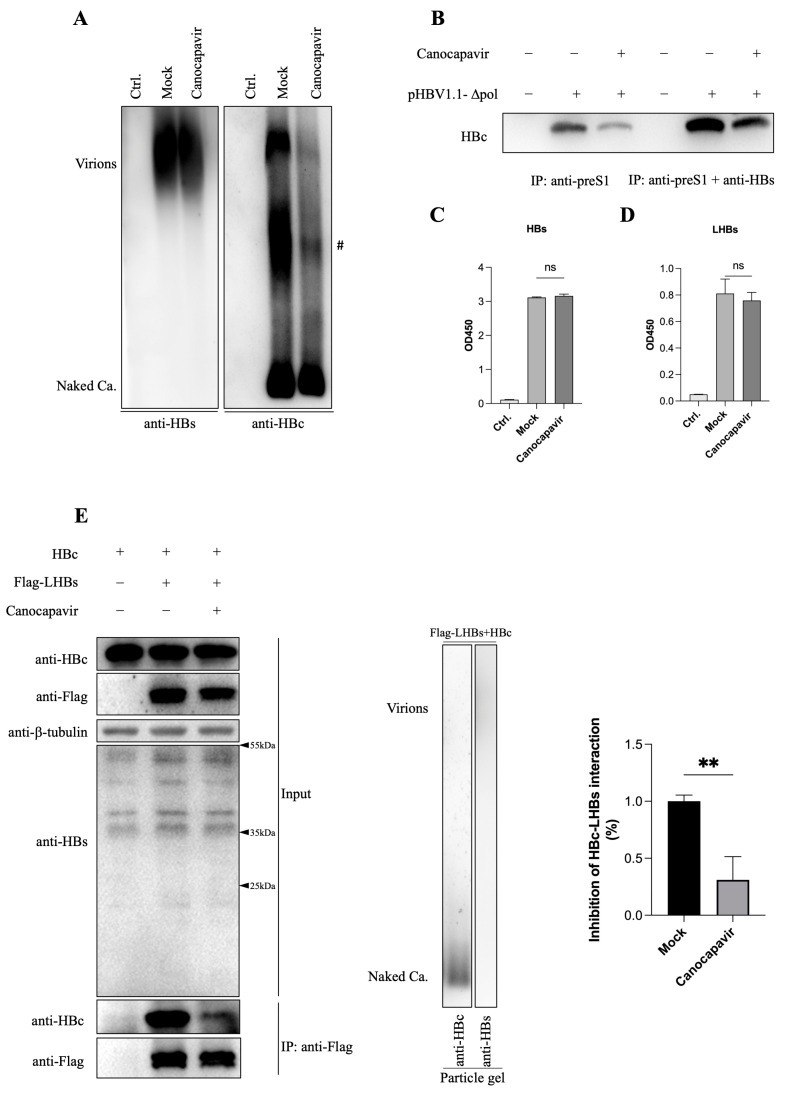
Canocapavir reduces the secretion of empty virions by interfering with the interaction between HBc and LHBs. (**A**) Huh 7 cells transfected with pHBV1.1-Δpol were mock-treated or treated with 3 μM Canocapavir for 4 days. Viral particles in concentrated cell culture media were resolved by native agarose gel electrophoresis, with the empty virions and naked capsids measured by immunoblotting with anti-HBs or anti-HBc (C1). (**B**) The empty virions in the cell culture media, as depicted in (**A**), were immunoprecipitated with either anti-preS1 or anti-preS1 plus anti-HBs, followed by Western blotting with anti-HBc (2A7). (**C**,**D**) The relative levels of HBs (**C**) and LHBs (**D**) in the cell culture media, as depicted in (**A**), were examined by ELISA. (**E**) Canocapavir treatment had a negative impact on the interactions between HBc and LHBs. Left, HEK 293T cells were co-transfected with plasmids expressing HBc and Flag-tagged LHBs at a 2:1 molar ratio, then mock-treated or treated with 3 μM Canocapavir. Cells transfected with the HBc plasmid alone served as control. A binding assay was performed by immunoprecipitation with anti-Flag and immunoblotting with anti-HBc (2A7). Middle, viral particles in the culture medium of transfected HEK 293T cells were resolved by particle gel assay as depicted in (**A**). Right, the relative density of immunoprecipitated HBc was calculated with data normalized to the level of Flag-LHBs. ** (*p* < 0.05) #, a non-specific band.

In addition to the complete virions, HBV-infected cells also secret a large quantity of virions that contain only the envelope protein and capsid. To explore the effect of Canocapavir treatment on the secretion of empty virions, Huh7 cells were transfected with an HBV replicon plasmid deficient in HBV polymerase expression (i.e., pHBV1.1-Δpol), then treated with Canocapavir. In spite of reduced egress of naked capsids, Canocapavir treatment also markedly decreased the amounts of empty virions from transfected cells, as compared with that of mock-treated cells (Figure 4A). We also compared the effect of Canocapavir on the secretion of empty virions with other type II CpAM (Figure S3). The envelopment of empty virions involves LHBs and SHBs proteins [29]. Virions in the cell culture of transfected Huh7 cells were precipitated with anti-preS1 and anti-S, or only with anti-preS1 antibody. Consistent with the result of the particle gel assay, less HBc was detected in the immunoprecipitated virions from Canocapavir-treated cells (Figure 4B). Nevertheless, the ELISA assay showed that there was no significant difference in the levels of extracellular SHBs and LHBs between mock- and Canocapavir-treated cells (Figure 4C,D). 

Among three surface proteins, the smallest SHBs alone is sufficient for empty virion secretion at a basal level, while the largest protein, LHBs, is essential for complete virion secretion and could stimulate empty virion secretion [29]. We next analyzed whether Canocapavir treatment has an impact on the interactions between HBc and LHBs. Cells were co-transfected with plasmids expressing HBc and Flag-tagged LHBs, then treated with Canocapavir. The co-IP assay suggested that LHBs could co-precipitate HBc, which, however was remarkably diminished in the presence of Canocapavir (Figure 4E). Taken together, these data suggested that Canocapavir treatment inhibits the envelopment of HBV virions, presumably by interrupting the interaction between HBc and LHBs.

### 3.5. Canocapavir Induces a Conformational Change at the HBc Linker Region

**Figure 5:**
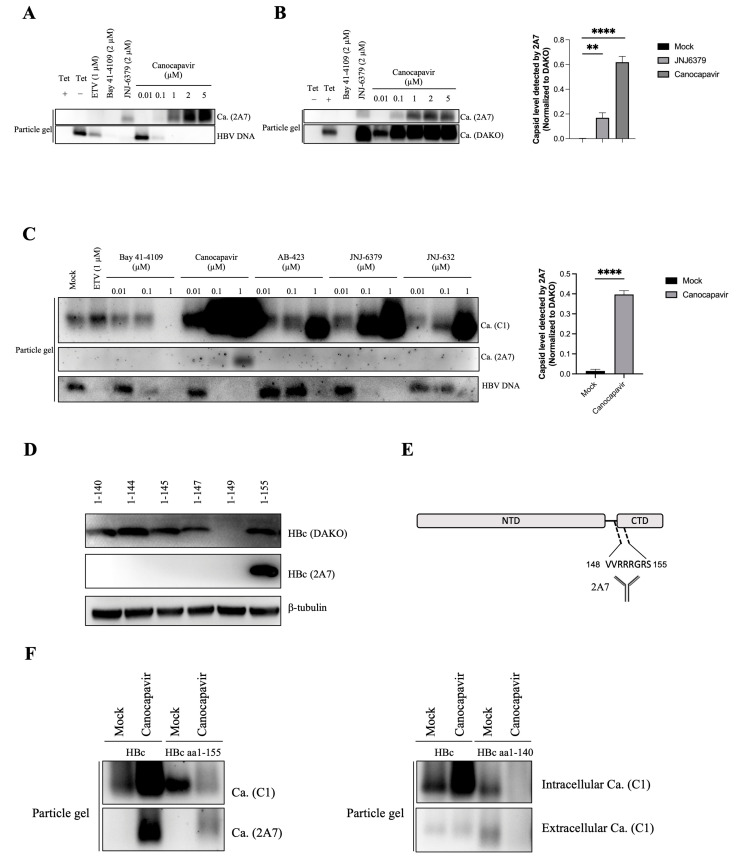
Canocapavir induces a conformational change of nucleocapsids. (**A**) HepAD38^Tet-off^ cells were cultured in a tetracycline-free medium and left untreated or treated with Bay 41-4109, JNJ-6379, or Canocapavir for 6 days at indicated doses. Intracellular capsids were separated by native gel electrophoresis and determined by immunoblotting with a monoclonal anti-HBc 2A7. Capsid-associated HBV nucleic acids were detected by a DIG-labeled HBV-specific probe. (**B**) HepG2-HBc^Tet-on^ cells were treated with Bay 41-4109, JNJ-6379, or Canocapavir at indicated doses for 2 days in the presence of 1 μg/mL tetracycline. Intracellular capsids were resolved by native gel electrophoresis and examined with anti-HBc 2A7 and DAKO, respectively (**left panel**). The relative density of 2A7-recognzing capsids was calculated in cells with or without the treatment of 2 μM Caonocapavir. Data were normalized to the level of capsids discernible to the DAKO anti-HBc (**right panel**). ** (*p* < 0.05), **** (*p* < 0.0001). (**C**) Left, HepG2.2.15 cells were treated with ETV, Bay 41-4109, Canocapavir, AB-423, JNJ-6379, or JNJ-632 at indicated concentrations, with intracellular capsids and the associated HBV nucleic acids determined as depicted in (**A**). Right, the relative capsid level detected by 2A7 was calculated as depicted in (**B**). **** (*p* < 0.0001). (**D**) Detection of capsids generated from C-terminally truncated HBc mutants by DAKO antibody and anti-HBc 2A7. C-terminally truncated HBc mutants, as indicated were transiently expressed in HEK 293T cells and subjected to Western blotting with 2A7 and the DAKO antibodies, respectively. (**E**) Schematic representation of the epitope recognized by anti-HBc 2A7. (**F**) HepG2 cells transiently expressing the full-length HBc, HBc aa1–155 (**left panel**), or HBc aa1–140 (**right panel**) were mock-treated or treated with 2 μM Canocapavir for 48 h. The intracellular capsids were resolved by native gel electrophoresis and detected by anti-HBc 2A7 or C1, respectively (**left panel**). Naked capsids in cell lysates and concentrated cell culture media were resolved by native gel electrophoresis and subjected to immunoblotting with anti-HBc C1 (**right panel**).

In order to investigate the molecular basis behind the interference of Canocapavir with the interaction of HBc and LHBs, we examined the configuration of HBc on the surfaces of capsids from Canocapavir-treated. Of particular interest, we found that, in contrast to capsids generated from mock or ETV-treated cells, capsids in HepAD38 ^Tet-off^ cells treated with Canocapavir could be specifically recognized by a monoclonal HBc antibody 2A7 (Figure 5A). The conformational change of capsids was recapitulated in a HepG2 strain with HBc expressed under the control of a Tet-On promoter. As shown in Figure 5B, the empty capsids could be probed by both 2A7 and DAKO anti-HBc antibodies in cells treated with JNJ-6379 and Canocapavir, while those in mock-treated cells could be only detected by the DAKO antibody. Compared to JNJ-6379 and other CpAMs, only Canocapavir induced such structural change of capsids at a dose of 1 µM in HepG2.2.15 cells (Figure 5C).

Anti-HBc 2A7 was raised against HBV precore peptide aa (–10) –152 and reported to bind HBc aa140–152 [28]. To map the precise target epitope of 2A7, we generated a variety of C-terminally truncated HBc mutants. As shown in Figure 5D,E, the DAKO antibody could detect all of these truncated HBc except for HBc aa1–149 due to its limited expression levels as a consequence of its failure to assemble into capsids and thus of its rapid degradation as subunits [11], the anti-HBc 2A7 reacted only with HBc aa1–155. HBc aa148–152 thus may represent a primary recognition site for 2A7. Therefore, it appears that Canocapavir treatment induced a conformational change of nucleocapsids, leading to full exposure of the C-terminal vicinity of the HBc linker region on the exterior of capsids.

In Canocapavir-treated cells, HBc aa1−155 was able to self-assemble into capsids with typical conformational change recognized by 2A7 (Figure 5F, left), indicating that this allosteric effect is independent of the rest flexible tail of CTD. Interestingly, the total capsids assembled from HBc aa1−155 were inhibited by Canocapavir as measured by the C1 antibody against an epitope nearby the spike region of HBc. The reduced capsid formation was also seen in Canocapavir-treated cells expressing HBc aa1−140 (Figure 5F, right) and several other HBc constructs truncated at the linker region (Figure S2).

Taken together, these results suggest that Canocapavir induces a conformational change at the HBc linker region on the surface of the capsid. Given the virological significance of the HBc linker, we posit that this allosteric effect may have great importance in the anti-HBV activity of Canocapavir.

### 3.6. The Binding Site of Canocapavir Is Located at the Hydrophobic (HAP) Pocket

**Figure 6:**
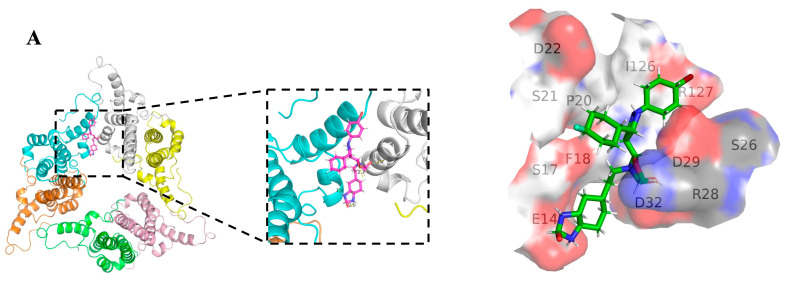

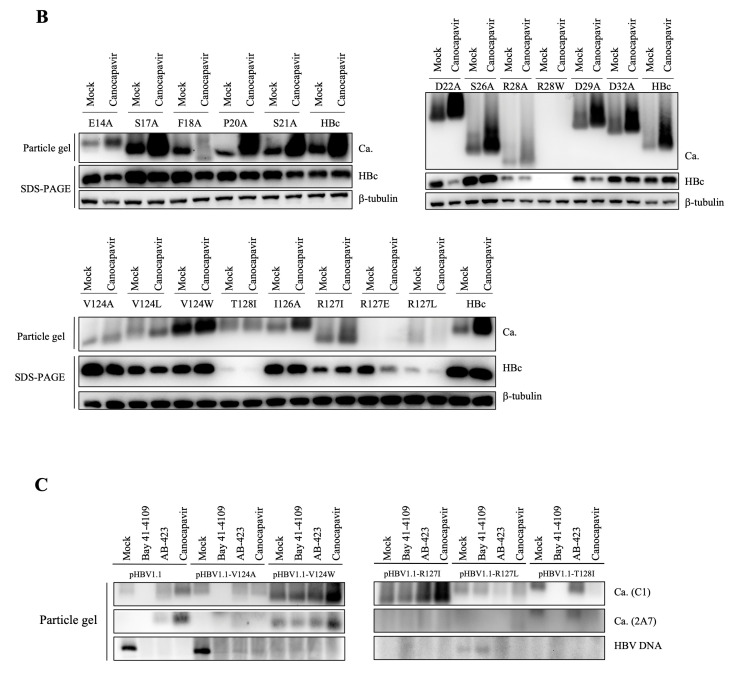
The binding site of Canocapavir is located at the HAP pocket. (**A**) Structural simulation and molecular docking analysis of the binding site of Canocapavir on HBc (PDB: 6J10). (**B**) HepG2 cells expressing HBc or indicated HBc mutants were mock-treated or treated with 2 μM Canocapavir for 48 h. Intracellular capsids were analyzed by native gel assay and immunoblotting with anti-HBc C1. The levels of total HBc expression were determined by Western blotting with anti-HBc 2A7. (**C**) HepG2 cells were transfected with pHBV1.1 or pHBV1.1-derived plasmids with indicated HBc gene mutations, left untreated or treated with 2 µM Bay 41-4109, 2 µM JNJ-6379, or 2 µM Canocapavir, respectively. Intracellular capsids were resolved by native gel electrophoresis and detected with anti-HBc 2A7 or C1, with associated HBV nucleic acids detected by hybridization with a DIG-labeled HBV-specific probe.

Previous structural studies have identified a hydrophobic pocket, designated as an HAP pocket, at the dimer-dimer interfaces near the C termini of HBc subunits. Accommodation of most CpAMs in this pocket enhances hydrophobic interaction between interfaces by filling the gaps [30,31]. To examine the possible binding sites of Canocapavir on HBc, the HBc structure (PDB: 6J10), represented as a trimer of dimers, and the structure of Canocapavir were subjected to energy minimization prior to the simulation of computational docking. A docking model with the highest binding affinity was shown in Figure 6A with the amino acids within 4Å of the ligand depicted, consistent with a typical HAP-targeting mode of CpAMs. According to the simulation results and previous studies [32], we constructed a series of HBc mutants on these contacting amino acids and measured the levels of HBc expression and capsid assembly in transfected HepG2 cells.

As shown in Figure 6B, except for the R127E incapable of capsid assembly and the R28W mutant that failed to be expressed, other HBc mutants were able to express HBc and assemble into capsids as well. Although charged amino acid residues, such as D22A, D29A, and D32A mutants of HBc, have a significant impact on capsid electrophoresis mobility [30], Canocapavir treatment did not apparently shift the capsid mobility. Of particular interest, we found F18A, R28A, V124A, R127L, and T128I mutants abolished the effect of Canocapavir-triggered intracellular accumulation of capsids. As R28 and R127 are at the bottom of the valley below spike near the dimer-dimer interface, and V124 is crucial residues at the wall of the HAP pocket, these data are consistent with the notion that Canocapavir targets HBc dimers in the vicinity of the HAP pocket.

HBc V124 residue is an important site for HAP pocket formation, and its substitution with amino acids bearing different hydrophobic side chains can significantly affect the assembly of capsid particles and interaction with CpAMs [32]. As illustrated in Figure 6C, V124W mutant of HBc assembled into more intracellular capsids in HepG2 cells comparted to WT HBc. The empty capsids formed in V124W-transfected cells could be recognized by anti-HBc 2A7, displaying a typical allosteric effect similar to that of Canocapavir treatment. Interestingly, Bay 41-4109 failed to abolish the capsid assembly of HBc V124W. V124A substitution, which resulted in a weaker contact energy in vitro [33], shifted the assembly equilibrium to fewer capsids but still supported the encapsidation of HBV pgRNA in cells. While the V124W substitution did not block the accumulation of capsids induced by Canocapavir, V124A was resistant to Canocapavir-triggered capsid accumulation. However, Canocapavir reduced the capsid-associated HBV DNA level of V124A mutant.

As illustrated in many crystal structures, R127 appears to have strong salt bridge interaction with D29 and D32, which likely enhances the stability of non-canonical dimer-dimer interaction [30]. We here identified that R127L but not R127I was resistant to the effect of capsid accumulation induced by Canocapavir. As common hydrogen bond was observed amongst CpAMs with T128 [32], of particular interest, Canocapavir remarkably diminished the capsid formation in cells transfected with HBV replicon carrying T128I mutation on HBc.

## 4. Discussion

HBV infection is a global health problem. Individuals with chronic HBV infection may progress to liver cirrhosis, hepatocellular carcinoma, or liver failure. The current antiviral drugs, including interferon (IFN) and nucleoside analogues (NAs), can effectively reduce viral load and prevent liver disease progression. However, while IFN therapy can achieve so-called functional cure in only a minority of patients, NAs fails to induce sustained off-treatment control of HBV replication and long-term use of NAs results in the emergence of drug-resistant HBV strains clinically [34,35,36]. Thus, novel antiviral strategies are still urgently needed.

HBV capsid assembly is a critical step in the propagation of virus and has become an attractive target for developing novel antiviral therapies. Currently, CpAMs under development fall into two classes according to their mode of action: either promoting core protein misassembly (type I) or accelerating assembly kinetics to form large number of empty capsids devoid of pgRNA (type II). Canocapavir has a type II CpAMs-like antiviral activity. Despite the fact that Canocapavir treatment increases intracellular capsids, the level of HBc expression was not affected, indicating that acceleration of capsid assembly kinetics is induced by Canocapavir binding. Assembly of HBV capsids is driven by the hydrophobic interaction of core proteins at dimer-dimer interface. Binding of CpAMs to a hydrophobic “HAP” pocket formed between the inter-dimer interface may strengthen the dimer-dimer interaction. Computational simulation suggested the docking site of Canocapavir on HBc was situated proximal to the HAP pocket. Through mutagenesis analysis, we found several mutants of HBc, especially residues at HBc dimer-dimer interface are critical for resistance to Canocapavir. Even though the HAP pocket can accommodate CpAMs with vastly different chemical cores, the interactions with certain residues, such as hydrogen bond with T128 and W102, are common amongst various CpAMs [32]. In this respect, it is noteworthy that the capsid assembly was disrupted in T128I mutant-transfected cells in the presence of Canocapavir. V124W is a HBc mutant which can assemble into more compact capsids by increasing buried hydrophobic surface and the strength of association energy proportionately [37,38]. HBc V124W is localized mainly in cytoplasm through immunofluorescence staining similar to subcellular distribution of HBc in Canocapavir-treated cells (Figure 2D). Intriguingly, the V124W mutant manifested a typical conformational change recognized by the anti-HBc 2A7, similar to that triggered by Canocapavir treatment (Figure 6C).

The secretion of HBV virions Initiates with the interaction between HBc and envelope proteins. Within the NTD and spatially located on the surface of capsid shell, a matrix binding domain (MBD) has been defined that is thought to interact with a short segment between R103 and S124 in the preS1 region of LHBs, the so-called matrix domain (MD), for complete virion formation [9,39]. It has been recently reported that HBc linker region could directly trigger the release of empty virions by interacting with the HBs protein, with LHBs being required to facilitate the secretion of large excess of empty virions [29]. Our study showed that the newly formed capsids in the presence of Canocapavir could be spatially detected by a monoclonal antibody 2A7 that recognizes HBc at the C-terminus of the linker region. Of note, we found that the interaction between HBc and LHBs was blocked in the presence of Canocapavir, presumably accounting for the decreased level of empty virions in the culture medium of Canocapavir-treated cells. Empty virions are secreted at more than 100-fold excess relative to complete virions in both cell culture and infected chimpanzees [6]. The LHBs on the surface of mature virions interacts with the sodium taurocholate co-transporting polypeptide (NTCP) on the hepatocyte surface through the preS1 region, establishing viral infection through the receptor-mediated endocytosis pathway. In this regard, it would be interesting to decipher the bio-significance of the excessive empty virions that are morphologically similar to the complete DNA-containing HBV virions.

Canocapavir treatment also led to decreased release of naked capsid from HBV- or HBc-transfected cells. Capsid particles are exported in a coatless state that is not fully understood. It was even posited that HBV might have a common ancestor with non-enveloped nackednaviruses [40]. Through loss-of-function and gain-of-function assays, we confirmed that the expression of Alix indeed assists in naked capsid secretion. Interestingly, overexpression of Alix abolished the inhibitory effect of Canocapavir on naked capsid egress. However, distinct from the previous study [27], no appreciable binding was observed between HBc and Alix in our co-immunoprecipitation assay. In transfected Huh 7 cells, Canocapavir treatment did not cause alterations in the spatial co-localization of HBc and endogenous Alix. It is previously reported that Rab33B and Atg5/12/16L1 are required for proper particle assembly, stability, and egress [41,42]. How naked capsids exploit the secretory pathway and how this allosteric effect induced by Canocapavir is involved in the regulation of capsid egress remain to be further elucidated.

In conclusion, Canocapavir demonstrates a robust antiviral activity and is a chemically and mechanistically unique type of capsid assembly modulator, thus representing a promising antiviral agent for treatment of chronic hepatitis B. Of particular note, our work strongly suggests the allosteric effect induced by Canocapavir treatment on HBc not only promotes the formation of capsid and disrupts the pgRNA encapsidation, but also reduces the capsid envelopment and secretion.

## Data Availability

Original data are available in notebooks, blots, computer, etc.

## Supplementary Materials

The following supporting information can be downloaded at: https://www.mdpi.com/article/10.3390/v15051195/s1, Figure S1: co-IP assay showed no interaction between HBc and Alix or Alix-Bro1; Figure S2: Canocapavir reduced the assembly of HBc constructs truncated at linker region; Figure S3: The effect of type II CpAM on the secretion of empty virions.
