# Supplementary material for: Canocapavir Is a Novel Capsid Assembly Modulator Inducing a Conformational Change of the Linker Region of HBV Core Protein
Source: Viruses. 2023 May 18;15(5):1195. doi: 10.3390/v15051195 (PMC10223407; doi:10.3390/v15051195)
Supplement: Supplementary file 1 [file viruses-15-01195-s001.zip › viruses-2375437-supplementary.pdf]

Supplementary 1

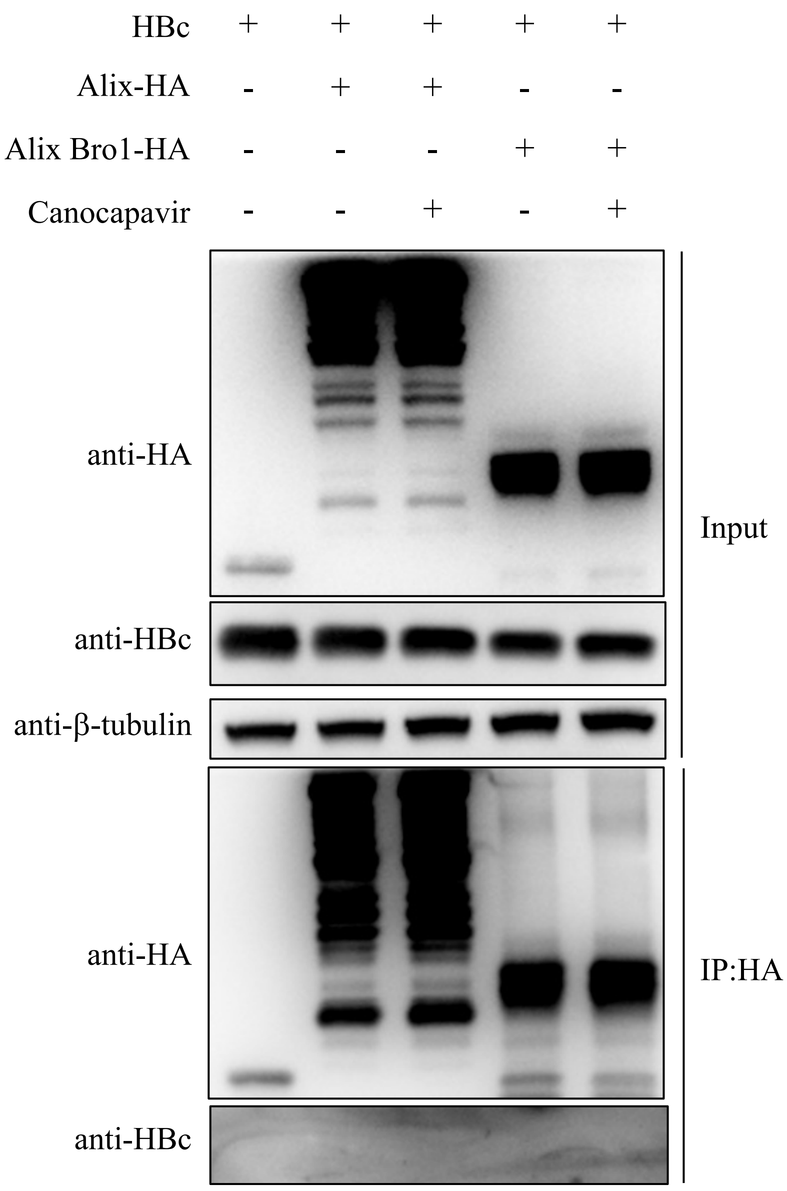

Figure S1: co-IP assay showed no interaction between HBc and Alix or Alix-Bro1. HEK 293T cells were co-transfected with plasmids expressing HBc and HA-tagged Alix or the Bro-1 domain at a 2:1 molar ratio, and then mock-treated or treated with 2 μM Canocapavir. Cells transfected with the HBc plasmid alone served as control. Binding assay was performed by immunoprecipitation with anti-HA and immunoblotting with anti-HBc (2A7).

Supplementary 2

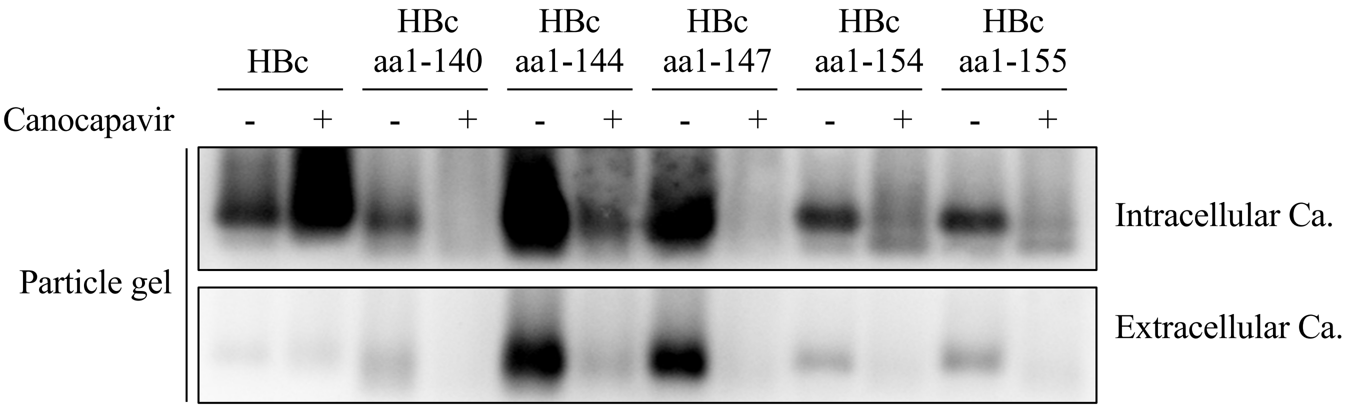

Figure S2: Canocapavir reduced the assembly of HBc constructs truncated at linker region. HepG2 cells transiently expressing the full-length HBc, HBc aa1–140, HBc aa1-144, HBc aa1-147, HBc aa1-154 or HBc aa1-155 were mock-treated or treated with 2 μM Canocapavir for 48 h. Naked capsids in cell lysates and concentrated cell culture media were resolved by native gel electrophoresis, and subjected to immunoblotting with anti-HBc C1.

Supplementary 3

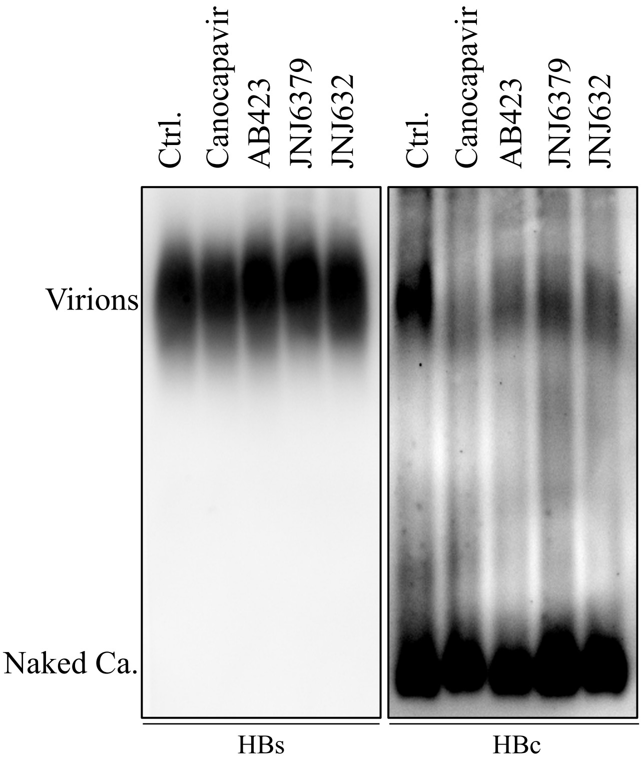

Figure S3: The effect of type II CpAM on the secretion of empty virions. Huh 7 cells transfected with pHBV1.1-Δpol were mock-treated or treated with 3 μM Canocapavir, AB423, JNJ6379 or JNJ632 for 4 days. Viral particles in concentrated cell culture media were resolved by native agarose gel electrophoresis, with the empty virions and naked capsids measured by immunoblotting with anti-HBs or anti-HBc (C1).
